# Functionality of technical working groups in enabling evidence-informed decision-making within Malawi's Ministry of Health: a cross-sectional qualitative study

**DOI:** 10.1186/s12961-023-00987-7

**Published:** 2023-06-06

**Authors:** Melody Sakala, Marlen Stacey Chawani, Isabel Kazanga-Chiumia, Hleziwe Hara, Leila Abdullahi, Dzinkambani Kambalame, Josephine Banda, Collins Mitambo, Anja Terlouw, Rose Oronje

**Affiliations:** 1 Malawi Liverpool Wellcome Trust Clinical Research Programme, Blantyre, Malawi; 2 Kamuzu University of Health Sciences, Blantyre, Malawi; 3 African Institute for Development Policy, Lilongwe, Malawi; 4 Malawi Ministry of Health, Lilongwe, Malawi; 5 Liverpool School of Tropical Medicine, Liverpool, England

**Keywords:** Technical advisory groups, Technical working groups, Functionality, Evidence, Decision-making, Health policy, LMIC

## Abstract

**Background:**

The roles and functionality of technical working groups (TWGs) in the health sectors vary across countries, still they aim to support government and ministries in formulating evidence-informed recommendations for policies and facilitate dialogue and alignment of activities among stakeholders within the health sector. Thus, TWGs have a role in enhancing the functionality and effectiveness of the health system structure. However, in Malawi, the functionality of TWGs and how they utilize research evidence to contribute to decision-making is not monitored. This study sought to understand the TWGs' performance and functionality in enabling evidence-informed decision-making (EIDM) in Malawi's health sector.

**Methods:**

A cross-sectional descriptive qualitative study. Data were collected through interviews, documents review and observation of three TWG meetings. Qualitative data were analysed using a thematic approach. The WHO-UNICEF Joint Reporting Form (JRF) was used to guide the assessment of TWG functionality.

**Results:**

TWG functionality varied in the Ministry of Health (MoH) in Malawi. The reasons for those perceived to be functioning well included meeting frequently, diverse representation of members, and that their recommendations to MoH were usually considered when decisions were made. For the TWGs that were perceived as not functioning well, the main reasons included lack of funding, periodic meetings and discussions that needed to provide clear decisions on the actions to be taken. In addition, evidence was recognized as important in decision-making, and research was valued by decision-makers within the MoH. However, some of the TWGs lacked reliable mechanisms for generating, accessing and synthesizing research. They also needed more capacity to review and use the research to inform their decisions.

**Conclusions:**

TWGs are highly valued and play a critical role in strengthening EIDM within the MoH. Our paper highlights the complexity and barriers of TWG functionality in supporting pathways for health policy-making in Malawi. These results have implications for EIDM in the health sector. This suggests that the MoH should actively develop reliable interventions and evidence tools, strengthen capacity-building and increase funding for EIDM.

## Background

Evidence-informed decision-making (EIDM) is a process in which high-quality, available evidence from research, local data, and patient and professional experiences are synthesized, disseminated and applied to decision-making in healthcare practice and policy [1]. Overall, there is a paucity of literature on how EIDM is used in policy processes and how this is enabled among technical working groups (TWGs). Whilst there is no universal definition of TWGs, they primarily consist of multidisciplinary individuals with relevant technical expertise to advise health policy-makers [2, 3]. TWGs' functionality and effectiveness may vary across different countries, sectors and programmes. Among other things, TWGs are mandated to provide independent evidence-informed advice to the government to assist in policy formulation [4], but they also function as a coordination structure for facilitating dialogue and alignment of activities among stakeholders within the health sector. Due to the multiple roles of TWGs, it is important to review the functionality and effectiveness of TWGs in fulfilling their defined mandates.

In Malawi, previous assessments of the functionality of TWGs revealed a plethora of TWGs in the health sector, which posed challenges to the functioning of the Ministry of Health (MoH) [5]. In 2015, the sector had 14 TWGs, 26 subcommittees, 13 task forces with 12 task teams (TTs) and expert groups, and it was recommended that six thematic working groups and six subsidiary steering committees should be established instead to align with WHO Health system building blocks. Whilst it was expected that TWGs would feed into the decision-making of the health sector working group (HSWG), there was a lack of clarity on how discussions from TWGs were processed and contributed to decision-making at higher levels, as only a few pertinent issues were put forward for discussion in the HSWG [5]. Instead, TWGs were noted to report mainly to the MoH Senior Management Team (SMT). Nevertheless, the TWGs were considered adequate as a platform for sharing project information but less effective as a coordination and decision-making structure.

Essentially, there is insufficient literature on the general functionality of TWGs for various programmes [2], and there are no international guidelines or standardized tools for these assessments.

Despite the gap and challenges in the structure and the functionality of TWGs in Malawi and several other countries, there is a lack of clarity on how research evidence is utilized in different TWG platforms to inform decisions and how the TWG discussions are processed and contribute to decision-making at higher levels [5-7]. This highlights the need to understand the functionality and effectiveness of TWGs in fulfilling their defined mandates, thus the aim of this study. The study also assessed the importance of the TWGs’ decision-making roles and their use of research evidence within the Malawi MoH.

## Methods

### Study context

This study was part of a 3-year (October 2019–October 2022) project called The Heightening Institutional Capacity for Government Use of Health Research (HIGH-Res) conducted in Kenya, Malawi and Uganda. The study was implemented to test interventions that strengthen the capacity of TWGs in enabling a sustained evidence use culture in the MoHs. This study focused on TWGs within the Ministry of Health (MoH) in Malawi, categorized as a low-income country in sub-Saharan Africa [8]. The Ministry is responsible for developing, reviewing and enforcing health-related policies and standards for the health sector [9]. The MoH performs its functions through 14 directorates, including a Planning and Policy Development Department and a Research Department. The MoH has made some effort to promote and strengthen EIDM by housing a knowledge translation platform (KTP-Malawi) within the Research Department [10]. The KTP’s mandate is to engage national-level policy-makers, researchers and implementers in coordinated production and use of research findings in the health sector, developing guidelines for evidence use in policy-making for researchers and policy-makers. In addition, the KTP conducts capacity-building in knowledge translation and utilization for policy-makers and researchers in EIDM [11].

### Study design

This study adopted a cross-sectional descriptive qualitative approach to allow for a deeper understanding of the performance and functionality of TWGs within the MoH, and the challenges and opportunities for strengthening EIDM within these structures. The study was conducted between January and February 2020.

### Study setting and target population

This was a national study involving top-level and mid-level decision-makers from the MoH, development partners, training institutions, and health researchers. Top-level decision-makers at the MoH consisted of the directors and deputy directors of various directorates and national programme managers. Mid-level policy-makers at the MoH comprised officers heading divisions, units and programmes. For development partner agencies and research and training institutions, the study targeted the leaders of these institutions.

### Sampling and sample size

A purposive sampling technique was employed to select participants from the MoH, research and training institutions, and development partners in Malawi to provide different, anonymized perspectives. A sample size of 57 participants was targeted. The participants were selected based on their professional positions, and this was guided by (i) the need to speak to individuals who possessed ample knowledge and experience of decision-making processes and systems within the MOH; (ii) the need to maximize triangulation of information sources; and (iii) the attainment of data saturation. The selection of study participants was made in consultation with the MoH. We interviewed 44 participants who gave consent, out of the total selected sample.

### Data collection

#### In-depth interviews

Data were collected through in-depth interviews (IDIs) with 44 key informants. The interviews were conducted between 21 January and 10 February 2020. Interviews were recorded in English and lasted on average 50 minutes. All interviews were recorded upon obtaining consent from the study participants. The data collection instruments used in the study included separate interview guides for MoH top-level policy-makers and mid-level officers, and interview guides for leaders of training institutions, research institutions and development agencies. Before data collection, the instruments were pretested on a few top-level policy-makers and researchers. Feedback from the pretest informed the revision and finalization of the tool.

#### Observations of TWG meetings

In addition to the individual IDIs, observations of three TWG meetings were conducted to understand the use of research evidence within the TWG decision-making structure. The meetings observed were opportunistic to the data collection period. A TWG observation guide was developed and used by the team during the observation of the meetings.

#### Desk review of policy documents and reports

A desk review of policy documents, research and other reports was also conducted to provide an understanding of the existing institutional structures and their functionality. The documents reviewed included relevant national strategies, guidelines and previous TWG assessment reports, among others.

### Data management and analysis

We adopted the WHO-UNICEF (United Nations Children's Fund) Joint Reporting Form (JRF) for our analysis. The form highlights six functionality indicators related to: the availability of Terms of Reference (TORs) and a legislative basis, frequency of meetings, areas of expertise for members, distribution of documents prior to meetings and disclosure of conflict of interest. The study employed three indicators from this framework and added some emerging critical themes related to EIDM. The research team transcribed data from interviews. Qualitative data were analysed using a thematic analysis approach coding. This involved immersion in the data, identification of codes, indexing the data to codes and identifying emerging themes and subthemes. The various meanings, attitudes and perceptions were interpreted while also drawing references from the wider literature.

### Ethics

The study was conducted in accordance with the ethical principles and guidelines in Malawi. Ethical clearance was obtained from the National Health Sciences Research Committee (NHSRC). Written consent was obtained from all participants before the interview, whilst verbal consent was sought from TWG chairs for observation of their TWG meetings. Each interviewee was assigned a code number that was used on all information collected.

## Results

### Interview response rate

Forty-four participants completed the in-depth semi-structured questionnaires (77% response rate). All participants interviewed had the opportunity to participate in the TWG meetings. Table 1 summarizes the interviews conducted.

**Table 1:** Interviews conducted

Type of respondent	Planned	Interviewed *n* (77%)
Top-level policy-makers at the MoH	19	15
Mid-level officers at the MoH	18	8
Leaders of development agencies	5	3
Leaders of research institutions	5	7
Leaders of training institutions	5	5
Technical staff in development agencies	5	2
Researchers	5	6
Total	57	44

### Functionality of TWGs

#### Legislative and administrative basis for the TWG

**Fig. 1:**
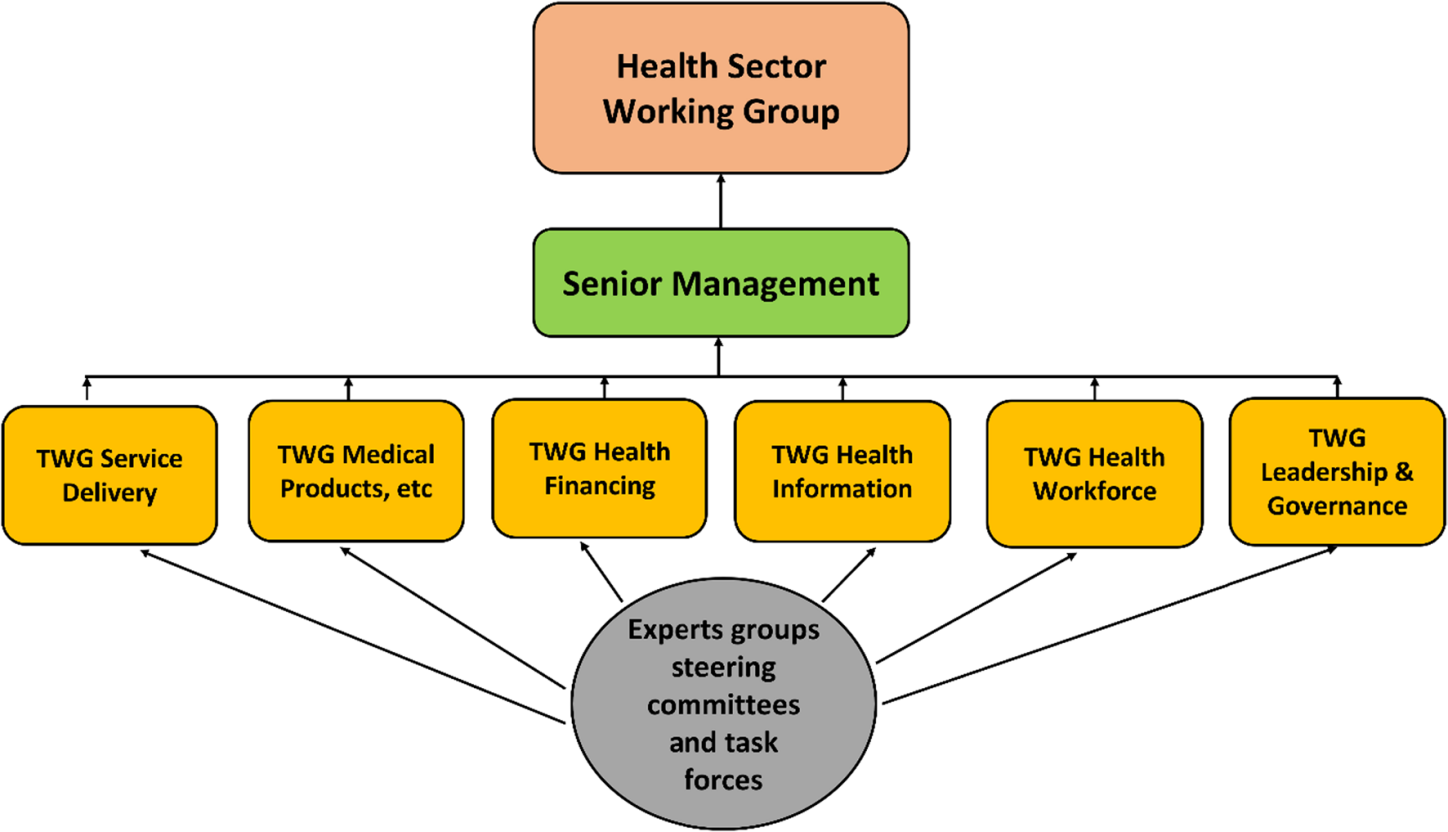
Ministry of Health and decision support structures. Source: MoH TWG Briefing Pack

A review of documents and key informant interviews found that TWGs are a crucial decision-making structure within the MoH. Figure 1 below depicts the official TWG structure within the MoH and how it links to other decision-making structures.

There are six TWGs formulated alongside the WHO health system building blocks: Service Delivery, Medical Products, Health Financing, Health Information, Health Workforce, and Leadership and Governance. The six TWGs report to the SMT, which reports to the HSWG that the Secretary of Health chairs. Under each of the six main TWGs, there are various subcommittees, task forces or expert groups generally referred to as sub-TWGs, where they report to the main TWGs. In this paper, TWGs will also refer to the sub-TWGs that operate under each of the six main TWGs. These TWGs are created based on tasks at hand, and so their roles and mandates are specific to each TWG (Table 2).

**Table 2:** Purpose of the six Ministry of Health Technical Working Groups

No.	TWG name	Purpose/mandate
1	Service Delivery	To provide input in and facilitate the review of the delivery of health services at all levels in line with identified health needs, the demand for health services and the affordability of the essential health package as well as professional standards for quality-of-service provision
2	Medical Products, Vaccines, Technologies and Infrastructure	To provide input in and facilitate the development of standards and specifications of pharmaceuticals to be included in the national Essential Drugs List, including the recommended antigens for the national Expanded Program on Immunization (EPI) as well as standards and specifications of medical equipment and health infrastructure
3	Health Financing	To provide technical input in and facilitate the development of a comprehensive but prioritized range of policy options for health system financing in Malawi for the medium and longer term
4	Health Information	To contribute to establishing an integrated health information system for the Malawi health sector that ensures the availability of accurate, reliable and timely information for planning, management and decision-making
5	Health Workforce	To provide technical input and advice on health workforce issues relevant to the implementation of the Health Sector Strategic Plan (HSSP), especially the implementation of the overall health workforce strategic plan
6	Leadership and Governance	To inform the MoH of changes that require appropriate responses/actions related to the health sector and MoH leadership and governance processes

During the interviews, it emerged that one of the primary directives of TWGs is to provide advice on technical matters, make recommendations, give policy guidance, endorsement and develop policy documents such as strategic plans and guidelines.

Another mandate for TWGs that emerged from the interviews is to convene all relevant partners implementing activities to share progress and challenges and to support the implementation of the Health Sector Strategic Plan through technical and financial assistance.

Other mandates of TWGs that emerged from the data included to manage collection of data, deliberate on the different sources of data (e.g. Department of Health Services), discuss and harmonize information for decision-making, respond to public health emergencies and epidemics in the country, and monitor policy implementation and service provision to ensure that the government is keeping its commitments.

General TORs were available for each of the six TWGs. The custodian of these TWGs was the Department of Planning and Policy which acted as the central coordinator within the MoH. Specific components included their purpose, functions, membership, frequency of meetings, reporting and delegation of authority. Each main TWG had specified indicators to track; however, no timelines were indicated for the activities.

#### Areas of expertise for members

A review of documents and key informant interviews established that the membership of the TWGs is multi-sectoral, diverse and clearly outlined in the existing TORs of each TWG. Broadly, the TWG membership comprises MoH staff from relevant departments, development partners, civil society, the private sector and academic institutions.

*The membership consists of highly technical people even though sometimes some members do not have knowledge and skills.* (Researcher, 7 February 2020)

….* Some memberships are appointed based on positions within institutions*. (Researcher, 6 February 2020)

Even though the roles and responsibilities of all members in the TWGs are elaborated in the TORs, the respondents outlined the following roles: the MoH leadership chairs TWG meetings and coordinates the meetings, researchers represent their institutions on TWGs as members and play the role of technical personnel, who often provide technical papers, reports and present data to TWGs.

A few researchers act as secretaries on their TWGs. In addition, development partners represent their institutions as members who provide technical advice to TWGs.

*To provide technical advice as a member and also secretary*. (Researcher, 30 January 2020)

In one instance, one development partner reported that they chair the Population and Development TWG. Some development partners are given opportunities to support hosting the TWGs based on MoH requests.

*Sometimes we support in hosting the TWGs, and this is based on request*. (Leader in development agencies, 30 January 2020)

#### Frequency of TWG meetings

Even though TWGs are scheduled to meet once every quarter according to their TORs, it was established that only some TWGs were able to meet regularly according to schedule. For instance, one TWG reported meeting only once in the past year, whereas some TWGs reported meeting only twice in the past year. Some TWGs only meet when there is a need, for example, during emergencies, and stop meeting when the emergencies have been resolved.

*Meetings depend on arising emergency issues*. (Top-level policy-maker, MoH, 11 February 2020)

*Frequency of meetings is a challenge*. (Technical staff of development agency, 20 January 2020)

*I do not know because I was only able to attend two meetings*. (Leader of a development agency, 20 January 2020)

Respondents indicated that the MoH sets the agenda of the TWG meeting with members' input. The agenda is often informed by arising issues and updates on annual programme implementation.

*Challenges in terms of what is put on the agenda/structure of the agenda is more an operational issues.* (Technical staff of development agencies, 30 January 2020)

Following the meetings, TWGs either submit reports or make presentations to the SMT of the MoH. In some cases, respondents indicated that reporting channels used include emails and WhatsApp groups.

#### Performance of TWGs

The data show that the perceived performance of TWGs varied. For the TWGs that were perceived to be performing their mandates well, this was mainly because their recommendations to the MoH were usually taken into consideration when decisions were made; they frequently met as required; there was a good representation of members; and the membership consisted of highly technical people.

*Technical vector control group, meet frequently in real time and clear agendas*. (Researcher, 30 January 2020)

For the TWGs that were perceived as not performing their mandates well, the main reasons included fewer meetings (less than the required quarterly meetings) and fragmented discussions that did not provide clear decisions on the actions to be taken. For such TWGs, their actions need to be implemented, and therefore, follow-up is not satisfactory.

*Most of the TWGs are performing fairly well, but there is a need for some improvements with learning from other countries that are doing well. *(Top-level policy-maker, MoH, 27 January 2020)

*Not taking actions, they meet but not translated to a complete, and implementing and follow-up from TWGs is not good.* (Technical staff in development agencies)

#### Funding for TWGs

Funding emerged as an important determining factor in TWG performance. Results show that development partners largely fund TWGs since the Ministry does not provide a budget for TWG activities. Respondents argued that TWGs still need to graduate from depending on partners for funding and coordinating their logistics. This can be incredibly costly since funds are needed to cover travel costs for members from other country districts.

*Funding is a challenge. External funding comes with conditions.* (Mid-level policy-maker, MoH, 6 February 2020)

It was further established that due to lack of funding, TWGs meet infrequently, and meetings are sometimes unplanned and shifted around. Ideally, MoH departments are required to budget for the activities of their TWGs. However, funding shortfalls are expected.

#### Research use in TWGs

Most top- and mid-level MoH officials and staff perceived that the MoH used research and data well or very well in their discussion. On the other hand, respondents outside the Ministry (i.e. researchers and leaders of research institutions and development partner agencies) alluded that the MoH perceived research as “very important” in decision-making. It was reported that TWGs use research to inform their decisions, raise awareness on emerging issues, exchange ideas and lessons, provide information on where resources are being spent in the health sector, and lobby partners for funding and technical support. Nevertheless, it was highlighted that even though evidence was perceived as central to decision-making, uptake of recommendations based on evidence was often affected by several factors such as political interests and financial constraints. An MoH official reported that:

*When we encounter a challenge or recognize a need, we welcome member partners to share best practices based on evidence.* (Mid-level policy-maker, MoH, 5 February 2020)

**Fig. 2:**
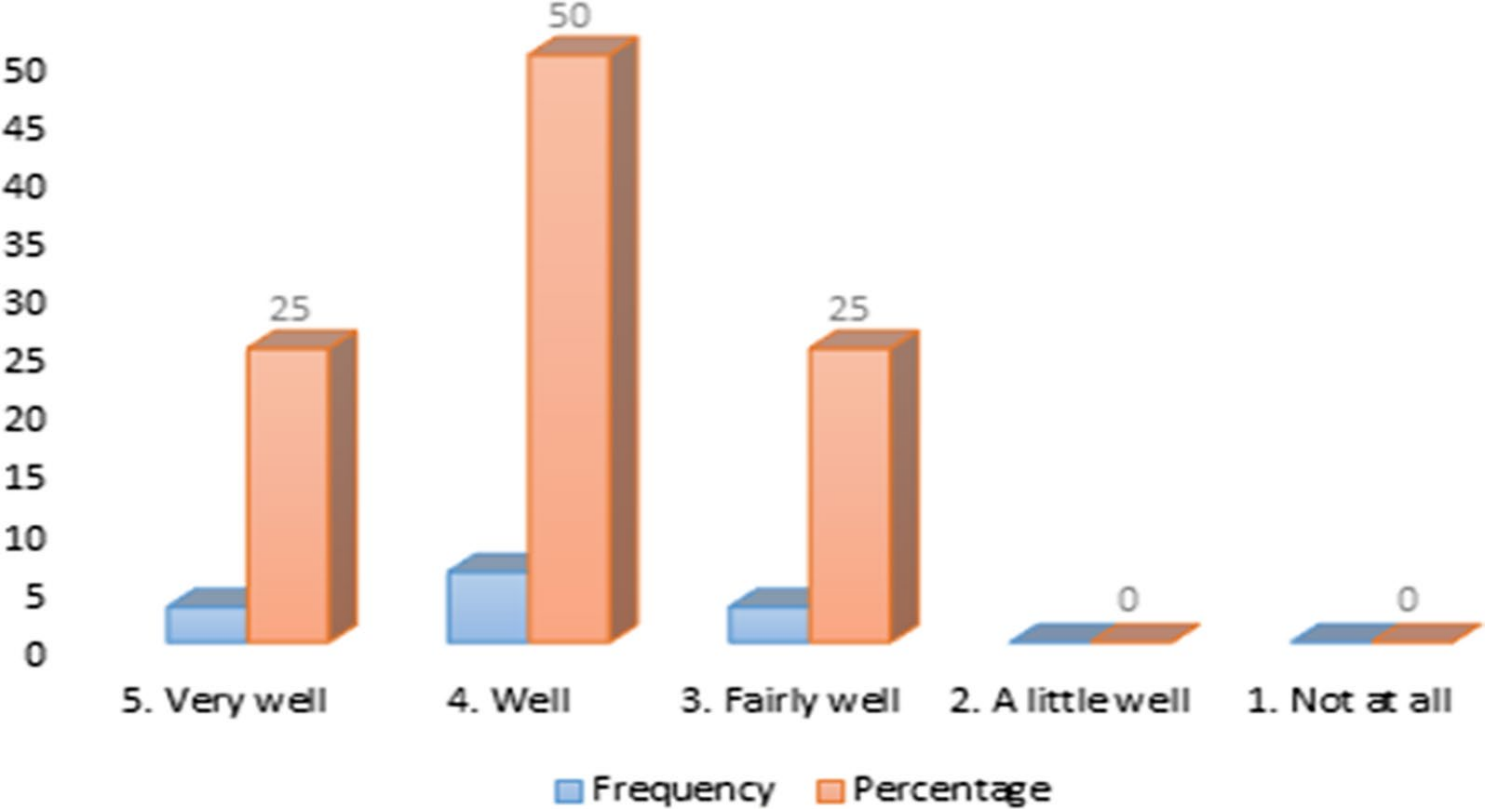
Extent to which structures discuss research and data for evidence-based decision-making

Figure 2 shows the perceived extent to which research was discussed to inform decision-making.

#### Researchers’ contributions to TWGs

Based on the researchers' perspectives, many TWGs have researchers actively involved in their meetings. Leaders of research institutions are also willing to formally require their researchers’ participation in TWGs in efforts to strengthen government decision-making. Researchers are also interested in being actively involved in TWG activities. Of those interviewed, researchers were involved in the following TWGs: malaria vector control, malaria in pregnancy, neglected tropical diseases, policy, HIV/AIDS, reproductive health, mental health, community health, tuberculosis, national immunization technical advisory groups (NITAGs), human resources, quality management, central hospital autonomy and pharmaceutical.

*TWGs and other high-level meetings with MoH act as a platform where research findings can be shared.* (Leader in research institution)

Study results show that researchers have substantial involvement in most of the existing TWGs. Most researchers participate in TWGs as representatives of academia, research institutions and development partners.

*Normally, the academia whose role is also to share relevant research with the TWGs*. (Top-level policy-maker, MoH, 30 January 2020)

*These are usually members from training institutions; they support the generation of evidence to inform policy.* (Top-level policy-maker, MoH, 30 January 2020)

Researchers actively involved in TWGs provide technical advice to inform policy, present data on ongoing studies to share emerging research on critical issues with TWGs, and conduct pilot studies and research needed for decision-making. In some TWGs, however, it was reported that even though research institutions are included as part of the membership of the TWG, no researchers were actively involved in the work of the TWG.

*Yes. They conduct pilot studies on issues, make presentations on ongoing studies. WHO has sponsored a study on emergency contracts for the department*? (Top-level policy-maker, MoH, 7 February 2020)

#### Existing capacity to use research evidence in TWGs

Regarding access to research evidence for decision-making, there was variation in reporting across members from different TWGs. Some TWG members reported having easy access to research evidence, for instance, through the MOH Research Department, research and training institutions, development partners and other members within the TWGs. On the other hand, some TWG members reported difficulties accessing research evidence and cited user fees for online journal databases as a major challenge. The participants were quoted as follows:

*We get health research through the research department but also health research institutions in the country, such as College of Medicine and [United Kingdom/United States] universities that have come with some findings that have really helped to change policy.* (Top-level policy-maker, 27 January 2020)

*It is not easy to access some research data as they have to pay, for example, to access some online journals.* (Top-level policy-maker, 30 January 2020)

Furthermore, the TWG members reported inadequate capacity to synthesize research evidence and apply it to inform decision-making. TWG members indicated that they mostly rely on external resources for synthesis of research evidence. TWG members also reported limited capacity to adapt research from elsewhere and apply it to local policy and programme needs. Some indicated that they had challenges in understanding and interpreting complex research that is often full of jargon.

*There is no capacity for members who can actually synthesize research.* (Top-level policy-maker, MoH, 28 January 2020)

They noted that even though the Public Health Institute of Malawi (PHIM) has researchers who could support them in translating complex research into easy-to-understand language, these researchers are few and therefore not able to meet the needs of all TWGs as far as research translation is concerned. They also reported that sometimes application of research findings is obstructed by resistance to adopt the evidence, especially when the evidence conflicts with the departmental practices. Some members explained as follows:

*Most research done does not respond to policy issues, hence it’s hard to apply every research.* (Top-level policy-maker, MoH, 30 January 2020)

*Resistance to adopt recommendations or research evidence, especially when the evidence provided is against current departmental practices.* (Top-level policy-maker, MoH, 30 January 2020)

## Discussion

TWGs in Malawi are well-established structures with various subcommittees which are not well coordinated. TWG members have diverse expertise; however, some need more capacity to synthesize and use evidence in decision-making. Irregular meetings affect TWG effectiveness. TWGs are poorly funded and donor-dependent. In addition, research is used in TWGs; however, uptake is affected by several factors. Overall, the findings of this study show that TWGs are a key decision-making structure within the MoH, whose main mandate is to provide advice on health systems technical matters, make recommendations, give policy guidance and develop policy documents. These findings are consistent with results from other studies [4, 5, 12]. Since TWGs are expected to provide independent and evidence-based guidance to the MoH in decision- and policy-making, integration of EIDM into TWGs is crucial to ensure effective decision- and policy-making [13].

The findings further show that the TWG structure for the health sector in Malawi is well defined and has become more streamlined over the years; the official number of TWGs reduced to six from 14 in 2015, as presented in the functional review of TWGs report [5]. Consequently, the number of sub-TWGs under each TWG is more controlled, ranging from two to 11 sub-TWGs, in contrast to over 39 subcommittees and task forces that existed in 2015. This can reduce duplication of activities and enhance efficiency in the operations of TWGs. Nevertheless, there is still a challenge in coordination, collaboration and sharing of information across different TWGs. It is thus necessary to strengthen coordination and communication mechanisms at different levels. Previous studies in other countries [4, 14-16] have stressed the importance of TWGs having a secretariat to coordinate their activities. A viable TWG structure with a secretariat and legally established TORs is essential for the effective functioning of TWGs [6, 17, 18]. In Malawi, the secretariat role for each TWG rests with the responsible MOH departments/programmes for the specific technical area, which was also the case in 2015 [5]. Furthermore, in line with previous recommendations to establish a Partners and Coordination Division within the Department of Planning and Policy Development (DPPD) to function as the HSWG secretariat [5], the DPPD has the responsibility to manage and coordinate the overall HSWG/TWG structure centrally.

Regarding the composition of the TWGs, this study revealed that membership for the health sector TWGs in Malawi was diverse and multidisciplinary. The availability of diverse and appropriate technical expertise in TWGs greatly influences their functionality, as it impacts the credibility of the recommendations made [2, 16, 18]. In addition, having researchers in TWGs provides the reliability of research used to inform local solutions [6] and is therefore vital in promoting EIDM. Previous studies observed a need for more relevant expertise from groups in most advisory groups in low- and middle-income countries (LMICs) [19]. For some TWGs in Malawi, the required technical expertise was lacking in discussions due to the delegation of the membership to junior personnel. Furthermore, membership is mostly through an invitation from the MoH, which may be influenced by the nature of the existing relationships between the MoH and the stakeholders, rather than technical expertise. [6]. Additionally, the reputation and integrity of members is also an important aspect to consider in the selection process [15, 20].

The number of meetings held per year is one of the indicators of the functioning of advisory groups such as TWGs [21, 22]. This study revealed that the meetings across all MOH TWGs were irregular despite the recommended quarterly time frame. The frequency of the meetings depended on the availability of resources. These findings are consistent with a systematic review of the functionality of NITAGs in Africa [19] where the number of TWGs who met annually was low. Furthermore, it was observed [6] that TWGs with adequate funding were associated with regular meetings and their deliberations were most likely to inform decision-making at high levels. Well-planned and well-resourced meetings have a major effect in the functionality of TWGs, as they drive the goals and objectives to discuss relevant health issues leading to recommendations. Specifically for low-income countries like Malawi, alternative ways of increasing the frequency of meetings should be explored. In this era of coronavirus disease 2019 (COVID-19), use of virtual platforms for meetings should be promoted to increase participation and ease financial constraints for TWGs without support. Physical TWG meetings would still be necessary, potentially held biannually, to provide opportunities for networking and relationship-building among stakeholders working in the sector, which is essential for good coordination of activities. Additionally, meetings need to be preplanned with enough notice given to participants rather than scheduling or announcing them ad hoc as previously recommended [15]. This is particularly important in promoting EIDM, as it may allow members to adequately prepare by reviewing, in advance, evidence that is useful to inform decisions. Regular meetings may be achieved by ensuring annual work plans have clear timelines for meetings and stakeholders identified to fund the meetings.

Inadequate funding emerged as a significant challenge affecting the functionality and sustainability of TWGs. Currently, funding is hugely dependent on donor aid. As observed by two global studies [6, 19], financial challenges noted in many African countries affect the functions and mandates of TWGs and their sustainability in the long run.

The use of research evidence in decision-making was valued highly and used by decision-makers within the MOH. Barriers to the use of evidence included lack of reliable mechanisms and capacity for generating, accessing, synthesizing and using research. Other study findings show similar barriers [16, 19]. In addition, uptake of recommendations based on evidence was affected by political interests and financial constraints. These findings resonate with Bell's [14] study on the effectiveness of TWGs in LMICs, where development of recommendations varied across groups and was hampered by lack of a systematic way to arrive at conclusions, as noted in Uganda, Senegal and Indonesia [6].

The study established variations in the capacity of TWGs to generate, synthesize and use research evidence, and this means that efforts to build technical capacity should further be strengthened. These results are consistent with the findings of other studies [6, 7, 14, 19]. Bell [6] further noted that time and publication languages were barriers to use of evidence by decision- and policy-makers, including TWGs. In Malawi, initiatives to strengthen individual and institutional capacity in research synthesis and evidence use are being explored. However, tracking and monitoring of these initiatives is lacking. Perhaps targeted capacity-building for motivated staff who can act as champions can be explored. To strengthen capacity within TWGs, other studies recommend visits to other NITAGs which can facilitate cross-learning, capacity-building training and a repository to access relevant materials for TWGs [6].

## Limitations

This work was based on key informant interviews with mostly MoH staff and TWG members who self-assessed the platforms, and this has potential for bias. However, their views were aligned with the reported findings from those working outside the MoH who were interviewed. Another limitation was that most literature reviewed focused on NITAGs, which might not be representative of the general functionality of TWGs. Nonetheless, TWGs mandates, operations and procedures are similar.

## Conclusion

TWGs are a key decision-making structure within the MoH in Malawi. While this is the case, TWGs lack reliable mechanisms for accessing research and for reviewing and synthesizing research to inform the decisions they make. TWGs are also inadequately funded and often rely on development partners, which affects their operations. This therefore calls for the MoH to urgently find sustainable ways of raising funds for the work of TWGs. TWGs also have weak capacity for synthesis of existing research, as well as in translating and communicating research effectively, both of which are critical for enabling EIDM. To strengthen TWG functionality, there is a need for urgent interventions that will enable them to conduct analysis of existing data, and review and synthesize existing research, to inform their decisions as and when needed. In addition, there is a need to institute within the MOH a viable TWG structure that has a sustainable financial mechanism to develop TWG monitoring tools and ensure provision of technical support. To gain the most out of the TWGs, future efforts by the MoH in low-income countries should focus on regular monitoring to improve functionality and track levels of recommendations used for decision-making and strengthen the integration of EIDM processes in these structures.

## Recommendations

We lay out the following recommendations to improve the functionality of TWGs:

MoH should ensure that the DPPD is equipped with adequate personnel and infrastructure to strengthen coordination and collaboration across the different TWGs.

It is therefore recommended that the TORs for each TWG be updated to incorporate roles that explicitly promote EIDM within the TWGs. In addition to strengthening the technical expertise in the TWGs, it is essential to strengthen the credibility of the decision-making process by ensuring rigour and transparency in the process.

Hence, there is a need to develop TWG membership selection guidelines to be used when nominating participants for TWGs in order to ensure the necessary range of expertise is included in the MoH TWGs, as was done for NITAGs in various countries.

In Malawi, to monitor and effectively strengthen functionality, the MoH should consider adapting the WHO-UNICEF JRF for TWG assessment. The tool can be used by the TWGs for annual self-assessment of their functionality to improve monitoring of their performance.

Malawi and other African governments should commit and support financial resources for the sustained functionality of TWGs and strengthening capacity-building initiatives in EIDM for TWGs within the MoH.

A representation of TWGs could benefit from a study tour to other countries with similar set-up so as to learn how to improve or maintain best practices.

## Data Availability

Applicable data generated or analysed in any way during this study are made available through the additional files. However, the recorded and transcribed notes are not made available to the public to ensure confidentiality of participants. All analysed data are available upon reasonable request from the corresponding author.
